# *Hydrangea medogensis* (Hydrangeaceae), a new species from Xizang (Tibet), China

**DOI:** 10.3897/phytokeys.279.203326

**Published:** 2026-09-10

**Authors:** Wen-Qi He, Wei-Lie Zheng, Fang-Yu Zhao, Kai-Xin Yin, Lian-Qiang Li, Kai-Hui Zhao, Fang-yuan Zhang, Zhi-hua Liao, Xiao-Zhong Lan

**Affiliations:** 1 School of Ecology and Environment, Xizang University, Lhasa, Xizang 850032, China School of Ecology and Environment, Xizang University Lhasa China; 2 Key Laboratory of Traditional Zang Medicine Resources Conservation and Utilization of Xizang Autonomous Region, Xizang Agriculture and Animal Husbandry University, Linzhi 860000, China Key Laboratory of Traditional Zang Medicine Resources Conservation and Utilization of Xizang Autonomous Region, Xizang Agriculture and Animal Husbandry University Linzhi China; 3 SWU-TAAHC Medicinal Plant Joint R&D Centre, School of Life Sciences, Southwest University, Chongqing 400715, China SWU-TAAHC Medicinal Plant Joint R&D Centre, School of Life Sciences, Southwest University Chongqing China

**Keywords:** *
Hydrangea
*, morphological analysis, new species, phylogenetic analysis

## Abstract

Based on field surveys, examination of herbarium specimens, and molecular phylogenetic analyses, the present study described a new species, *Hydrangea medogensis***sp. nov**. (Hydrangeaceae), from Medog County, Xizang Autonomous Region, China. Phylogenetic analyses indicated that *H. medogensis* was placed in Clade 1 as a distinct lineage, sister to the remainder of the clade. Morphologically, the new species is most similar to *H. stylosa* but can be distinguished by the following key characteristics: fertile flowers with a globose calyx tube constricted at the mouth; calyx teeth triangular, occasionally oblong-ovate, 0.6–1 mm long; adaxial leaf surface covered with two types of soft hairs (long and short); ovary more than 4/5 inferior, nearly completely inferior; and seeds with a very narrow wing around the margin. The taxonomic status of the new species was established based on integrated morphological and molecular evidence.

## Introduction

*Hydrangea* Gronov. ex L. *s.l*. belongs to Hydrangeaceae, subfamily Hydrangeoideae, and is one of the most important understory components in warm-temperate and tropical forests in East Asia. The main life forms of the genus include shrubs, herbs, and climbing shrubs (Samain et al. 2010; De Smet et al. 2015; Yang et al. 2024). In early studies, the genus was classified in the tribe Hydrangeeae of Hydrangeaceae and comprised approximately 70 species, distributed mainly in East and Southeast Asia and from the southeastern United States to Central America and western South America (Wei and Bartholomew 2001). However, subsequent studies revealed that *Hydrangea* s.s. was not a monophyletic group (Samain et al. 2010). Consequently, De Smet et al. (2015) proposed the concept of *Hydrangea* s.l. (*sensu lato*), treating the former genera *Broussaisia* Gaudich., *Cardiandra* Siebold & Zucc., *Decumaria* L., *Deinanthe* Maxim., *Dichroa* Lour., *Pileostegia* Turcz., *Platycrater* Siebold & Zucc., *Schizophragma* Siebold & Zucc., and *Hydrangea* s.s. as sections within *Hydrangea* s.l. This concept was subsequently supported by Yang et al. (2024).

During the field survey of the Fourth National Chinese Medicine Resource Census in the Xizang Autonomous Region conducted from 2013 to 2015, unidentified specimens of a *Hydrangea* species were collected in Medog County, Nyingchi City, Xizang Autonomous Region. The locality was revisited in July–August 2025, and additional herbarium and molecular materials were collected. Through careful examination of relevant taxonomic literature (Hooker and Thomson 1858; Wei and Bartholomew 2001) and herbarium specimens, combined with phylogenetic analysis, this taxon was identified as a new species of *Hydrangea*. The new species is morphologically similar to *Hydrangea stylosa* Hook.f. & Thomson. Based on field surveys, examination of herbarium specimens, and molecular evidence, the new species is formally described in the present study.

## Materials and methods

### Morphological assessment

Three specimens from different individuals were collected in Medog County, Nyingchi City, Xizang Autonomous Region, in 2015 and 2025. The morphological description of the new species was based on field observations of living plants, careful examination of color photographs, and dissection and measurement of pressed specimens. Morphological comparisons between the new species and similar species were conducted with reference to relevant literature (Hooker and Thomson 1858; Wei and Bartholomew 2001) and herbarium specimens. Voucher specimens are deposited at KUN (Kunming Institute of Botany, Chinese Academy of Sciences, China, Yunnan, Kunming) and XZE (Research Institute of Xizang Plateau Ecology, China, Xizang, Bayi Town, Linzhi). Herbarium acronyms follow Index Herbariorum (Thiers 2026).

### Phylogenetic analysis

Based on the literature (De Smet et al. 2015; Zhang et al. 2021; Uemachi et al. 2026) and GenBank search results, internal transcribed spacer (ITS) sequences of 55 samples were downloaded from GenBank for phylogenetic analysis, with *Philadelphus mexicanus* Schltdl., *P. pekinensis* Rupr., *Deutzia crassifolia* Rehder, *D. sieboldiana* Maxim., and *D. wardiana* Zaik. as outgroups (Table 1). Fresh silica-dried leaf material of *H. medogensis* collected in the field was sent to NovoGene (Beijing, China; https://cn.novogene.com/) for sequencing, and raw data with approximately 5× genome coverage were obtained using the Illumina NovaSeq platform.

**Table 1.** GenBank accession numbers and vouchers for the samples used in this study.

**Species**	**Voucher**	**ITS**	**Length**
*Hydrangea acuminata* Siebold & Zucc.	–	LC882542	591 bp
*Hydrangea acuminata* Siebold & Zucc.	–	KX065313	597 bp
*Hydrangea angustipetala* Hayata	141	LN830358	611 bp
*Hydrangea anomala* D.Don	1198	LN830355	611 bp
*Hydrangea arborescens* L.	D.Atha 8406 (NY)	KP120062	596 bp
*Hydrangea arborescens* L.	84	LN830406	610 bp
*Hydrangea aspera* Buch.-Ham. ex D.Don	40	LN830401	617 bp
*Hydrangea aspera* Buch.-Ham. ex D.Don	1551	LN830363	613 bp
*Hydrangea chinensis* Maxim.	1241	LN830356	610 bp
*Hydrangea chinensis* var. *yayeyamensis* (Koidz.) T.Yamaz.	–	AB377206	670 bp
*Hydrangea chungii* Rehder	248	LN830398	611 bp
*Hydrangea formosana* Koidz.	148	LN830361	611 bp
*Hydrangea glabripes* Rehder	194	LN830379	615 bp
*Hydrangea heteromalla* D.Don	87	LN830407	610 bp
*Hydrangea hirta* (Thunb.) Siebold	–	LC883905	590 bp
*Hydrangea hydrangeoides* (Siebold & Zucc.) Bernd Schulz	–	LC704900	797 bp
*Hydrangea integrifolia* Hayata	1197	LN830354	612 bp
*Hydrangea involucrata* Siebold	–	LT838926	615 bp
*Hydrangea involucrata* var. *idzuensis* Hayashi	–	LT838925	615 bp
*Hydrangea kawagoeana* Koidz.	–	AB377205	669 bp
*Hydrangea kawagoeana* var. *grosseserrata* (Engl.) Hatus.	–	AB377203	669 bp
*Hydrangea kawakamii* Hayata	–	LT838930	614 bp
*Hydrangea liukiuensis* Nakai	–	AB377201	670 bp
*Hydrangea longifolia* Hayata	–	LT838923	614 bp
*Hydrangea longipes* Franch.	75	LN830404	616 bp
*Hydrangea luteovenosa* Koidz.	196	LN830380	612 bp
*Hydrangea macrophylla* (Thunb.) Ser.	–	LC882516	592 bp
*Hydrangea macrophylla* f. *otaksa* (Siebold & Zucc.) E.H.Wilson	JBRI-10-002	GU983033	690 bp
*Hydrangea moellendorffii* Hance	–	LC613004	767 bp
*Hydrangea paniculata* f. *velutina* (Nakai) H.Ohba	–	LC612993	775 bp
*Hydrangea paniculata* Siebold	–	KX065325	597 bp
*Hydrangea petiolaris* Siebold & Zucc.	–	KX065298	597 bp
*Hydrangea quercifolia* W.Bartram	98	LN830408	611 bp
*Hydrangea radiata* Walter	231	LN830396	610 bp
*Hydrangea robusta* Hook. f. & Thomson	1114	LN830409	617 bp
*Hydrangea sargentiana* Rehder	81	LN830405	616 bp
*Hydrangea scandens* (L.f.) Ser.	202	LN830381	611 bp
*Hydrangea scandens* subsp. chinensis E.M.McClint.	QI-0641	KF559183	629 bp
*Hydrangea seemannii* L.Riley	74	LN830403	613 bp
*Hydrangea serrata* (Thunb.) Ser.	1653	LN830368	611 bp
*Hydrangea serrata* f. acuminata (Siebold & Zucc.) E.H.Wilson	18	LN830377	611 bp
*Hydrangea serrata* f. *buergeri* (Siebold & Zucc.) Nakai	–	KX065312	597 bp
*Hydrangea serrata* f. *fertilis* Nakai	–	KX065309	597 bp
*Hydrangea serrata* var. *angustata* (Franch. & Sav.) H.Ohba	–	LC883892	591 bp
*Hydrangea serrata* var. serrata	–	LC883898	591 bp
*Hydrangea serrata* var. *yesoensis* (Koidz.) H.Ohba	–	LC882540	592 bp
*Hydrangea sikokiana* Maxim.	1687	LN830370	610 bp
*Hydrangea strigosa* Rehder	237	LN830412	613 bp
*Hydrangea stylosa* Hook. f. & Thomson	210	LN830386	610 bp
*Hydrangea stylosa* Hook.f. & Thomson	207	LN830384	610 bp
*Hydrangea medogensis* sp. nov.	542624250727068LY	PZ487614	599 bp
*Hydrangea medogensis* sp. nov.	542624250801133LY	PZ487615	599 bp
*Philadelphus mexicanus* Schltdl.	Ph	LN830393	614 bp
*Philadelphus pekinensis* Rupr.	108	LN830347	611 bp
*Deutzia crassifolia* Rehder	B.Yang 76-2 (KUN)	KP120128	594 bp
*Deutzia sieboldiana* Maxim.	K.Kano 7912 (PE)	KP120121	594 bp
*Deutzia wardiana* Zaik.	Qinghai-Tibet Expediation Team 4444 (PE)	KP120126	594 bp

For the chloroplast genome, assembly was performed using GetOrganelle v1.7.7.1 (Jin et al. 2020), followed by automatic annotation using the online platform CPGAVAS2 (http://47.96.249.172:16019/analyzer/annotate; Shi et al. 2019). Manual verification and refinement were conducted in Geneious Prime v2024.0.5 (Kearse et al. 2012). The annotated results were visualized using Chloroplot (https://irscope.shinyapps.io/Chloroplot/; Zheng et al. 2020). Inverted repeat/single-copy (IR/SC) boundary regions were analyzed using IRscope (https://irscope.shinyapps.io/irapp/; Amiryousefi et al. 2018). In addition, chloroplast genomes of seven other *Hydrangea* species were retrieved from NCBI for comparison (Table 2) to evaluate IR expansion/contraction patterns.

**Table 2.** Source information of chloroplast genomes.

**Species**	**chloroplast**
*Hydrangea chinensis* Maxim.	PQ593891
*Hydrangea macrophylla* (Thunb.) Ser.	NC_085353
*Hydrangea platyarguta* Y.De Smet & C.Granados	OL405445
*Hydrangea petiolaris* Siebold & Zucc.	OR268926
*Hydrangea luteovenosa* Koidz.	NC_035662
*Hydrangea febrifuga* (Lour.) Y.De Smet & C.Granados	MW928534
*Hydrangea serrata* (Thunb.) Ser.	KY412464
*Hydrangea medogensis* sp. nov.	PZ509095
*Hydrangea medogensis* sp. nov.	PZ509096

For the ITS sequences, because of the low sequencing depth, a reference-guided assembly strategy was adopted: ITS sequences of congeneric species were downloaded from the NCBI database as reference baits, and raw reads were screened and mapped using BLASTN v2.17.0+ (Altschul et al. 1990). Positive reads were extracted and merged into single-end reads using Seqtk v1.5-r133 (https://github.com/lh3/seqtk), followed by *de novo* assembly using SPAdes genome assembler v4.2.0 (Bankevich et al. 2012; parameters: --careful, --only-assembler, -k 21,33,45). The resulting contigs were processed using ITSx v1.1.3 (Bengtsson-Palme et al. 2013) to identify and extract the complete ITS region, which was then aligned with reference sequences and manually verified in Geneious Prime v2024.0.5 (Kearse et al. 2012). The 5' and 3' ends of the ITS alignment were trimmed to remove primer sites and ambiguously aligned regions in Geneious Prime v2024.0.5 prior to phylogenetic analysis.

All sequences were aligned using MUSCLE v3.8.31005 (Edgar 2004) and manually adjusted in PhyDE-1 v0.9971 (Müller 2005). The best-fit nucleotide substitution model was determined using Modeltest v3.7 (Posada and Crandall 1998) under the Akaike Information Criterion (AIC). Following this procedure, the general time reversible (GTR) model was selected as the best model. Maximum likelihood (ML) analysis was conducted using RAxML-HPC (Stamatakis 2006) under the GTR model, with 100 bootstrap replicates to assess branch support. Bayesian phylogenetic trees were constructed using MrBayes v3.2.7 (Huelsenbeck and Ronquist 2001), starting from random trees with four Markov chain Monte Carlo (MCMC) chains and sampling every 100 generations for a total of 1,000,000 generations. Tree visualization was performed using FigTree v1.4.4 (Rambaut 2008), and the tree was subsequently edited in Adobe Illustrator 2023.

### Conservation assessment

The preliminary conservation assessment was conducted following the IUCN Red List Categories and Criteria (IUCN 2024). The extent of occurrence (EOO) and area of occupancy (AOO) were calculated based on the three known occurrence points. The AOO was estimated using the standard 2 × 2 km grid cell size following the methodology of the geospatial conservation assessment tool GeoCAT (Bachman et al. 2011).

## Results

### Taxonomic treatment

#### Hydrangea medogensis

Taxon classificationPlantaeCornalesHydrangeaceae

X.Z.Lan, W.L.Zheng & W.Q.He sp. nov.

FCAECAF3-A7F3-5978-822D-5990D97A7590

urn:lsid:ipni.org:names:77396492-1

##### Type.

**Figure 1.**
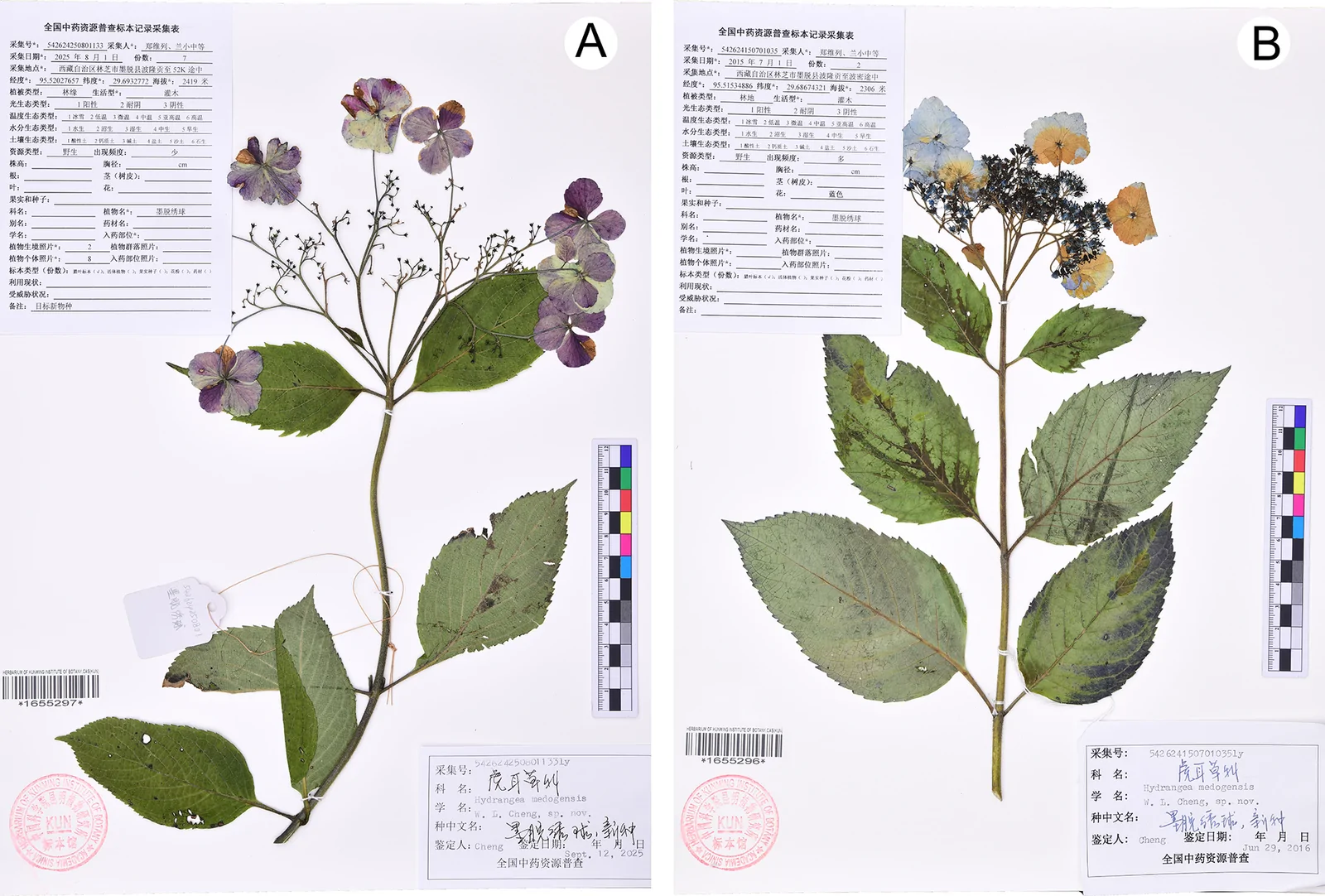
*Hydrangea medogensis* X.Z.Lan, W.L.Zheng & W.Q.He. **A**. Holotype; **B**. Paratype specimens.

**Figure 2.**
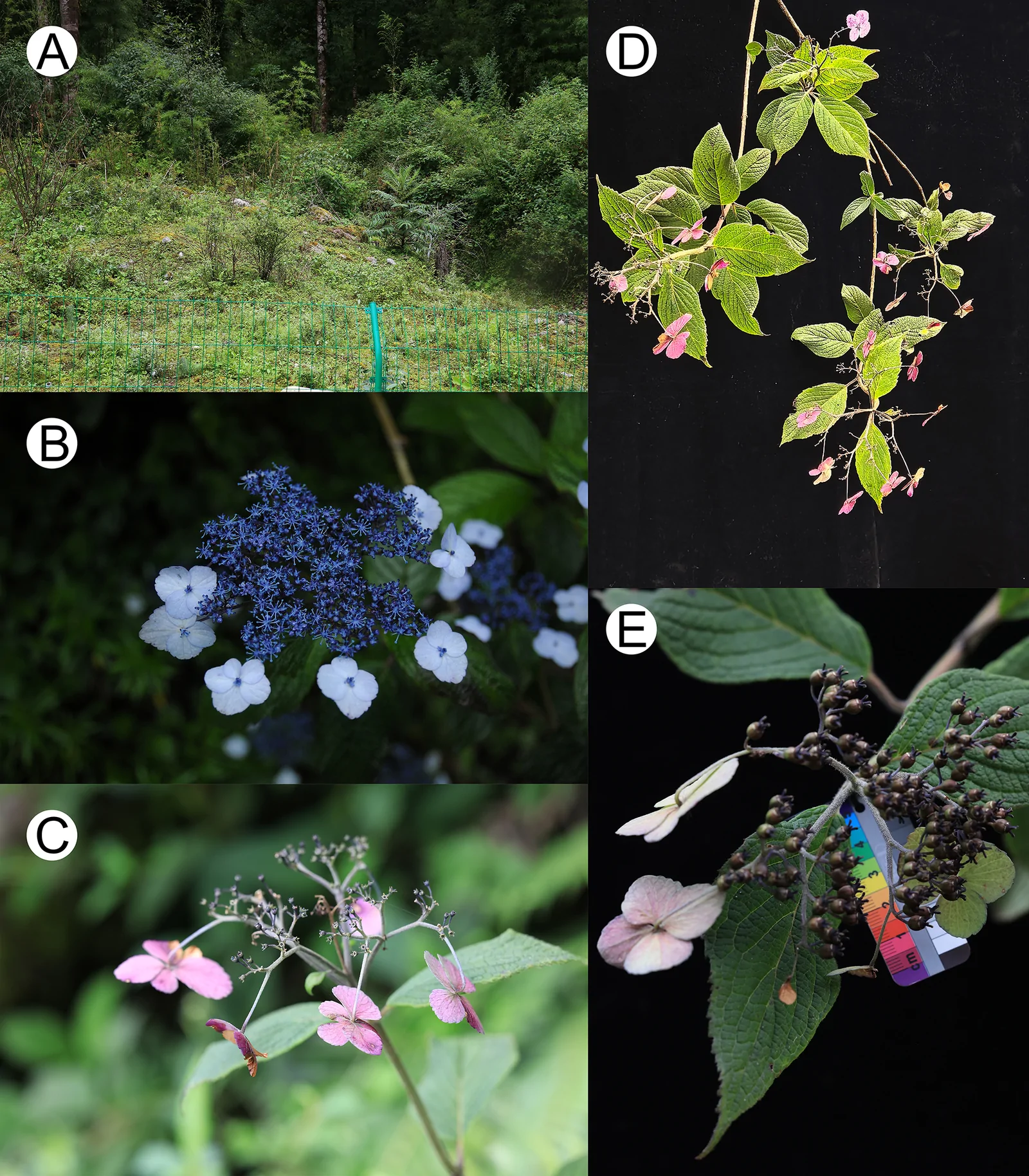
*Hydrangea medogensis* X.Z.Lan, W.L.Zheng & W.Q.He. **A**. Habitat; **B**. Flowering branch; **C**. Sterile flowers and young fruits; **D**. Branchlet; **E**. Fruiting branch.

**Figure 3.**
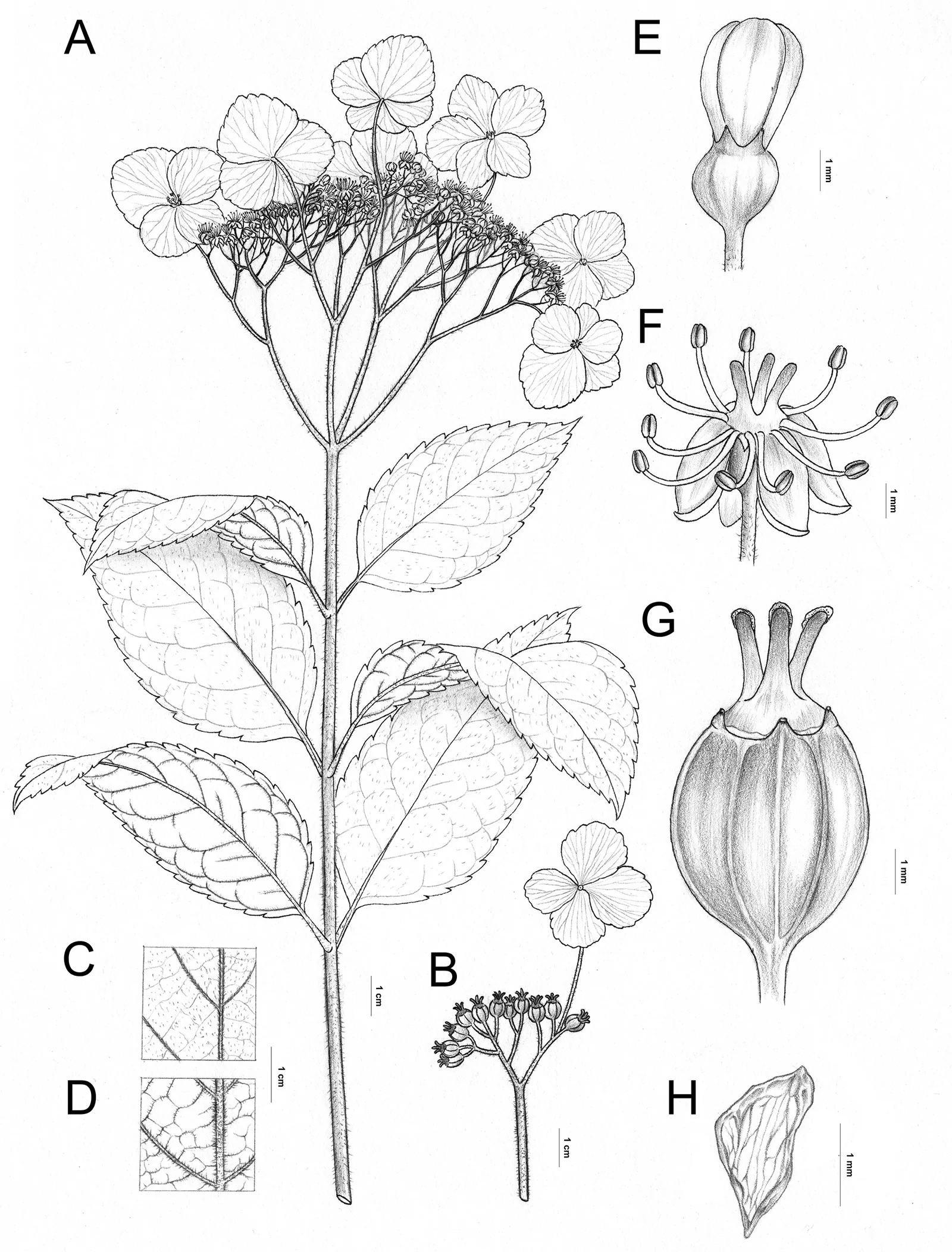
*Hydrangea medogensis* X.Z.Lan, W.L.Zheng & W.Q.He. **A**. Habit; **B**. Fruiting branch; **C**. Adaxial surface of leaf; **D**. Abaxial surface of leaf; **E**. Flower bud; **F**. Flower; **G**. Fruit; **H**. Seed (drawing by Wenqi He).

**Figure 4.**
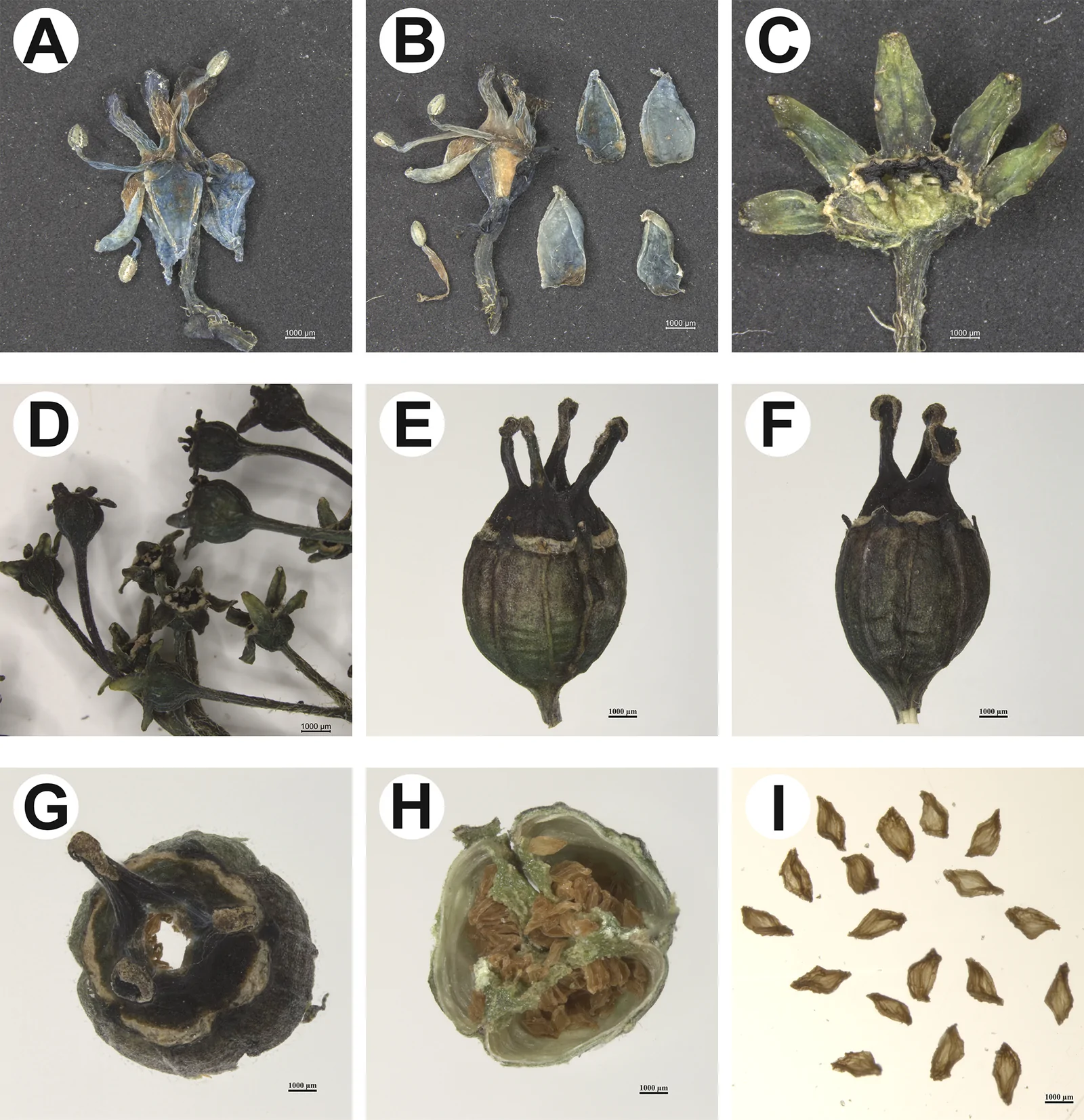
*Hydrangea medogensis* X.Z.Lan, W.L.Zheng & W.Q.He. **A**. Fertile flower; **B**. Dissected fertile flower; **C**. Dissected young fruit; **D**. Young fruit; **E, F**. Fruit; **G**. Apical view of fruit; **H**. Dissected fruit; **I**. Seeds.

China, Xizang Autonomous Region • Nyingchi City, Medog County, Zhamo Route, alt. ca. 2420 m, 29°41'36"N, 95°31'13"E, 1 August 2025, *Xiaozhong Lan, Weilie Zheng, Lianqiang Li & Wenqi He 542624250801133LY* (holotype: KUN!, barcode 1655297; isotype: XZE!). (Figs 1, 2, 3, 4; Table 3).

**Table 3.** Morphological comparisons between *Hydrangea medogensis* and *Hydrangea stylosa*.

**Characters**	***Hydrangea medogensis***	***Hydrangea stylosa***
Calyx	Fertile flowers with calyx tube subglobose, calyx opening constricted, calyx teeth triangular, occasionally oblong-ovate, 0.6–1 mm long	Fertile flowers with calyx tube campanulate, calyx opening tubular, calyx teeth ovate or suboblong, 1–1.5 mm long
Leaf	Adaxial surface of leaf covered with long and short hairs	Adaxial surface of leaf glabrous
Ovary	Ovary more than 4/5 inferior, nearly completely inferior	Ovary ca. 2/3 inferior
Seed	Seed narrowly winged around margin	Seed minutely winged at one or both ends

##### Diagnosis.

*Hydrangea medogensis* is morphologically similar to *H. stylosa*, but differs in having ovaries more than 4/5 inferior (vs. ca. 2/3 inferior), leaves with adaxial surface covered with long and short hairs (vs. nearly glabrous or glabrous on both surfaces), fertile flowers with calyx tube subglobose, calyx opening constricted, calyx teeth triangular, occasionally oblong-ovate, 0.6–1 mm long (vs. calyx tube campanulate, calyx opening tubular, calyx teeth ovate or suboblong, 1–1.5 mm long), and seeds narrowly winged around margin (vs. minutely winged at one or both ends).

##### Description.

Shrub, 1–1.5 m tall. One-year-old branchlets sparsely villous; two-year-old branchlets grayish-white, glabrescent. Leaves papery, elliptic or ovate-elliptic, 5.8–13 × 2.2–7 cm, apex shortly acuminate, base cuneate or subrounded, margin sparsely to densely serrate, teeth glandular at apex; adaxial leaf surface sparsely covered with two types of hairs (long and short, similar in texture), midvein densely puberulent; abaxial leaf surface villous only on veins; secondary veins 6–8 on each side of midvein, slightly curved, minor veins sparsely reticulate, abaxially slightly prominent; petiole 1–2.3 cm, covered with villous hairs, especially on the abaxial side. Inflorescence a corymbose cyme, terminal, with a peduncle 0.5–4.3 cm long, inflorescence 7–13 cm in diam., apex truncate; branches three, subequal, with the first-order branch of the central branch often lower, covered with yellowish puberulent hairs. Sterile flowers present; sepals (3)4–5, pale pink-purple to pale blue-purple, broadly ovate or rhombic-ovate, unequal, 1–2.4 × 1–2.4 cm, margin coarsely serrate, rarely entire, abaxially with puberulent hairs on veins; petals blue, minute, 1.5–2 mm, caducous. Fertile flowers with calyx tube subglobose; opening constricted, calyx teeth triangular, occasionally oblong-ovate, 0.6–1 mm, apex obtuse and gland-tipped. Petals blue, oblong-ovate, 2.5–3.5 mm, apex gland-tipped, conspicuous when dry, incurved in bud, reflexed after anthesis; petals free, base truncate. Stamens 8–10, unequal, the shorter ones 1/2 to 2/3 the length of the longer ones, the longer ones distinctly longer than petals; anthers suborbicular, 3-angled, ca. 0.8 mm, blue. Ovary from 4/5 inferior to nearly completely inferior; styles 3(4), short and thick, 2 mm long in fruit, expanded at base; stigmas small, capitate. Capsule subglobose, glabrous, ca. 5 mm long and 3.5 mm wide excluding styles, apical projection ca. 1 mm long, ca. 1/5 as long as the capsule, dehiscing apically among styles when mature. Seeds brownish, elliptic or oblong, 1.0–1.2 × 0.5–0.6 mm, with long reticulate venation, surrounded by a very narrow wing.

##### Phenology.

Flowering from June to July and fruiting from August to September.

##### Etymology.

The epithet indicates the type locality, i.e., Medog area, Xizang, China.

##### Vernacular name.

mò tuō xiù qiú (Chinese pronunciation); 墨脱绣球 (Chinese name).

##### Distribution and habitat.

*Hydrangea medogensis* was found in Medog County, Nyingchi City, Xizang (Tibet) Autonomous Region, China. This new species grows in the understory of broad-leaved forests and in shrub thickets at forest margins at elevations of 2200–2500 m.

##### Conservation status.

This species is currently known from three localities in Medog County. Each population comprises approximately 20–30 individuals, and the habitats are susceptible to disturbance or destruction. Based on this analysis, the extent of occurrence (EOO) was estimated to be ca. 13 km^2^ and the area of occupancy (AOO) was ca. 8 km^2^ (2 × 2 km grid). However, given that the species is known from only three localities and further field surveys are needed to assess its exact distribution, this species is provisionally assessed as Data Deficient (DD) following the IUCN Red List Categories and Criteria (IUCN 2024).

##### Additional specimen examined.

China • Xizang Autonomous Region: Nyingchi City, Medog County, Gedang Township, alt. ca. 2240 m, 29°27'53"N, 95°48'26"E, 27 July 2025, Xiaozhong Lan, Weilie Zheng, Lianqiang Li & Wenqi He 542624250727068LY (paratype: XZE!) • ibid., along road from Bolungong to Bomi, alt. ca. 2306 m, 29°41'23"N, 95°30'52"E, 1 July 2015, Xiaozhong Lan, Lianqiang Li & Fangyu Zhao 542624150701035LY (paratype: KUN!, barcode 1655296; XZE! ).

### Molecular phylogeny

**Figure 5.**
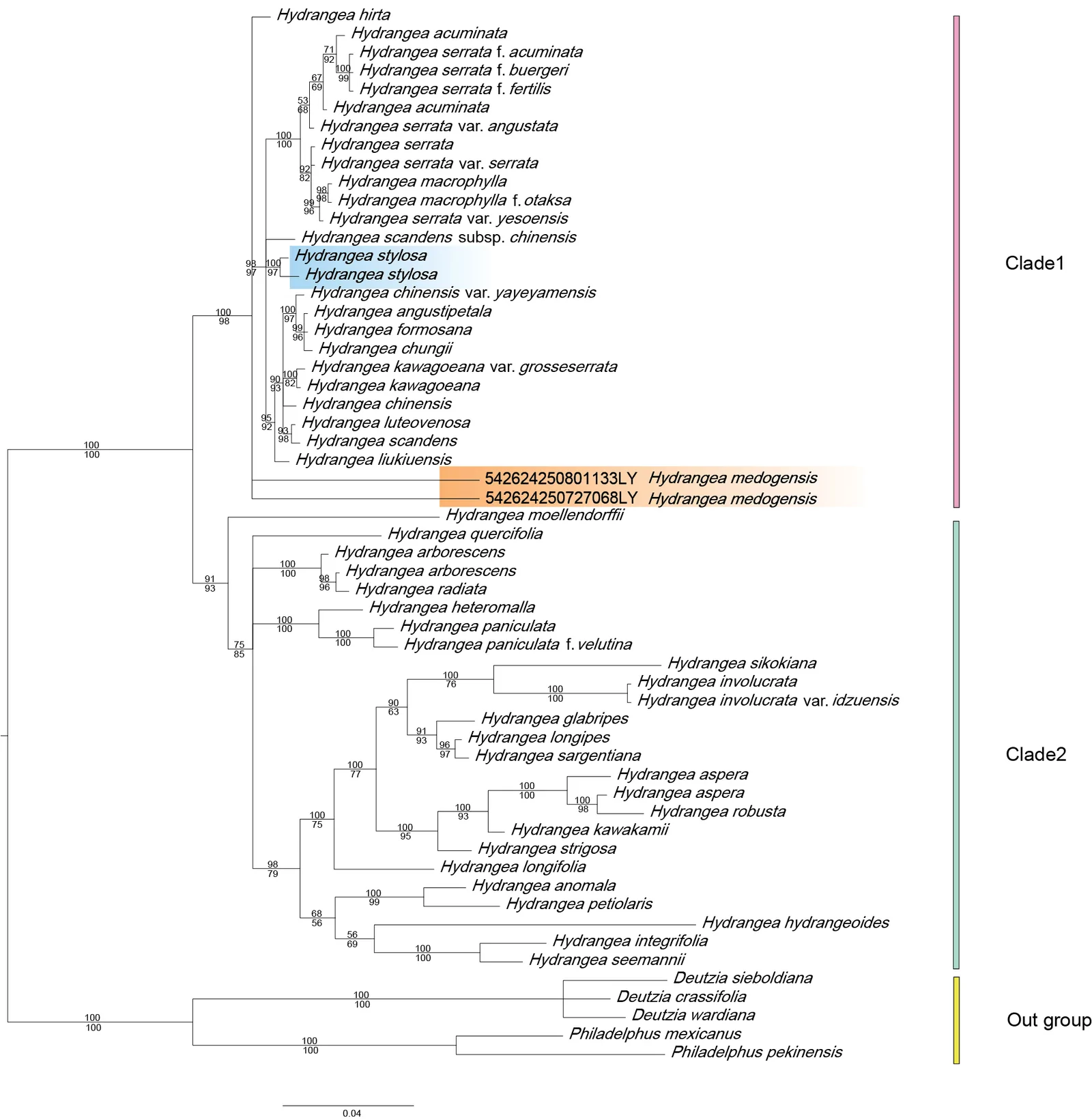
Bayesian consensus tree of *Hydrangea medogensis* and related species based on ITS sequences. The tree was constructed using the ITS sequences. Numbers below the branches represent maximum likelihood (ML) bootstrap support values, and numbers above the branches represent Bayesian posterior probabilities. *Hydrangea medogensis* is highlighted in orange.

Bayesian and maximum likelihood (ML) analyses produced similar phylogenetic topologies (Fig. 5). The phylogenetic results revealed that *Hydrangea* was divided into two major clades. The two samples of the new taxon formed a distinct terminal clade. Both samples were placed in Clade 1, supporting their conspecific status. *Hydrangea medogensis* was sister to the remainder of Clade 1.

### Chloroplast genome structure

**Figure 6.**
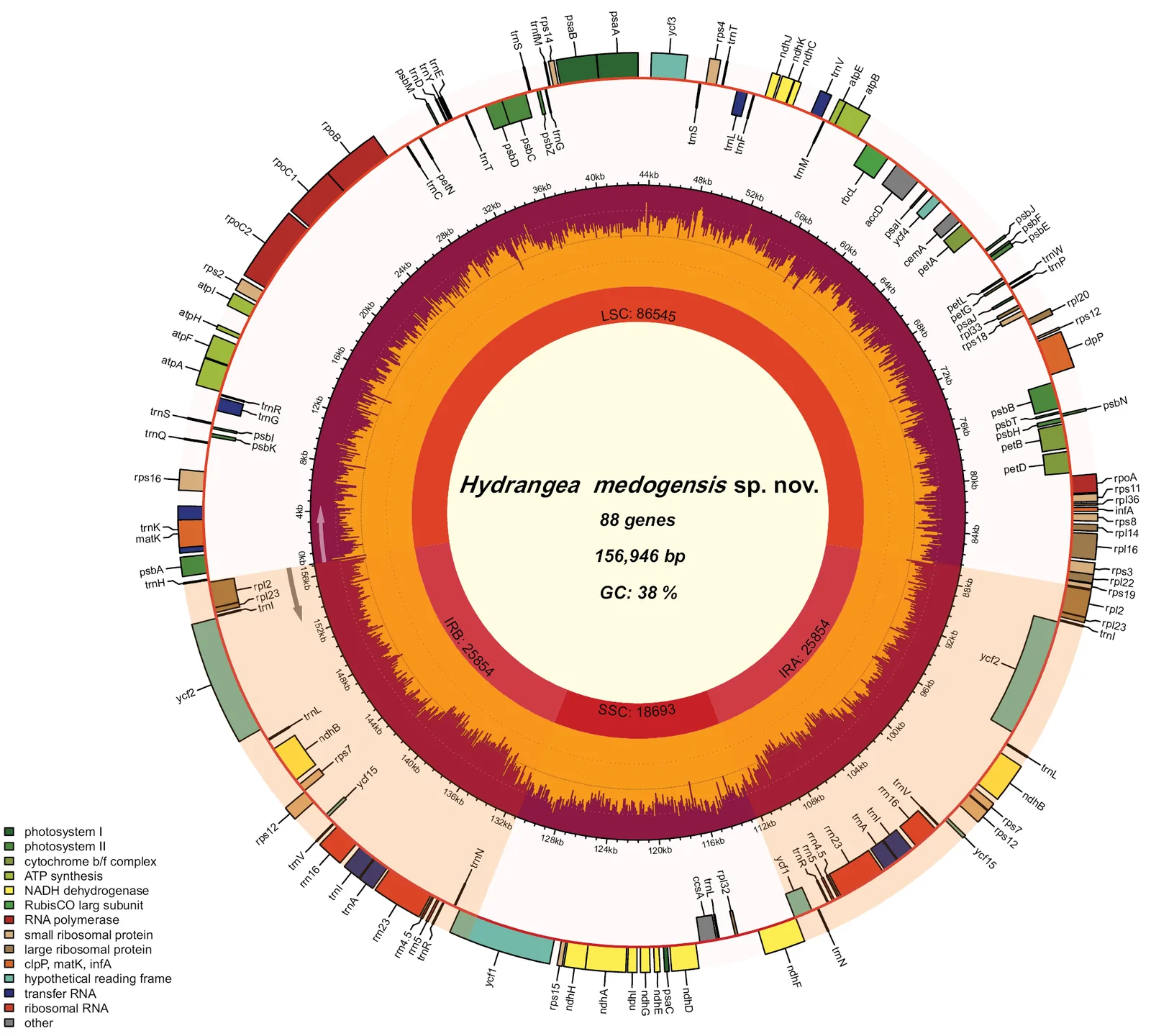
Gene map of the *Hydrangea medogensis* plastome. The circular map shows the typical quadripartite structure, with the large single-copy (LSC) and small single-copy (SSC) regions separated by two inverted repeats (IRa and IRb). Genes are color-coded by functional categories as indicated in the legend. Genes drawn inside the circle are transcribed clockwise, while those outside are transcribed counter-clockwise. The inner circle represents the GC content.

The complete chloroplast genome of *Hydrangea medogensis* was a circular molecule with a total length of 156,946 bp, exhibiting the typical quadripartite structure of angiosperm chloroplast genomes (Fig. 6). The genome comprised a large single-copy (LSC) region of 86,545 bp, a small single-copy (SSC) region of 18,693 bp, and two inverted repeat (IRa and IRb) regions of 25,854 bp each. The GC content was 38%, with the inner circle of the map showing the typical uneven distribution pattern among the four regions.

A total of 132 genes were encoded in the chloroplast genome, including 87 protein-coding genes, 37 tRNA genes, and eight rRNA genes (Table 4). Seventeen genes were duplicated in the IR regions, comprising all four rRNA genes (*rrn4.5S*, *rrn5S*, *rrn16S*, and *rrn23S*), six tRNA genes (*trnA-UGC*, *trnI-GAU*, *trnL-CAA*, *trnN-GUU*, *trnR-ACG*, and *trnV-GAC*), and seven protein-coding genes (*ndhB*, *rpl2*, *rpl23*, *rps7*, *ycf1*, *ycf2*, and *ycf15*). Genes were classified by function into categories related to photosynthesis, self-replication, and others, annotated with different colors in the circular map (Fig. 6), and transcribed in both clockwise and counter-clockwise directions.

**Table 4.** Gene composition of the *Hydrangea medogensis* chloroplast genome. Genes are categorized by functional groups. Duplicated genes in the inverted repeat (IR) regions are indicated with copy numbers.

**Category of genes**	**Group of genes**	**Name of genes**
Genes for photosynthesis	Subunits of ATP synthase	*atpA*, *atpB*, *atpE*, *atpF*, *atpH*, *atpI*
Genes for photosynthesis	Subunits of NADH-dehydrogenase	*ndhA*, *ndhB×2*, *ndhC*, *ndhD*, *ndhE*, *ndhF*, *ndhG*, *ndhH*, *ndhI*, *ndhJ*, *ndhK*
Genes for photosynthesis	Subunits of cytochrome b/f complex	*petA*, *petB*, *petD*, *petG*, *petL*, *petN*
Genes for photosynthesis	Subunits of photosystem I	*psaA*, *psaB*, *psaC*, *psaI*, *psaJ*
Genes for photosynthesis	Subunits of photosystem II	*psbA*, *psbB*, *psbC*, *psbD*, *psbE*, *psbF*, *psbH*, *psbI*, *psbJ*, *psbK*, *psbL*, *psbM*, *psbN*, *psbT*, *psbZ*
Self-replication	Large subunit of ribosome	*rpl14*, *rpl16*, *rpl2×2*, *rpl20*, *rpl22*, *rpl23×2*, *rpl32*, *rpl33*, *rpl36*
Self-replication	Small subunit of ribosome	*rps11*, *rps12*, *rps14*, *rps15*, *rps16*, *rps18*, *rps19*, *rps2*, *rps3*, *rps4*, *rps7×2*, *rps8*
Self-replication	DNA dependent RNA polymerase	*rpoA*, *rpoB*, *rpoC1*, *rpoC2*
Genes for photosynthesis	Subunit of rubisco	*rbcL*
Other genes	c-type cytochrom synthesis gene	*ccsA*
Other genes	Envelop membrane protein	*cemA*
Other genes	Maturase	*matK*
Other genes	Protease	*clpP*
Other genes	Subunit of Acetyl-CoA-carboxylase	*accD*
Other genes	Translational initiation factor	*infA*
Unknown	Conserved open reading frames	*ycf1×2*, *ycf2×2*, *ycf3*, *ycf4*, *ycf15×2*

Photosynthesis-related genes included six ATP synthase subunits (*atpA*, *atpB*, *atpE*, *atpF*, *atpH*, and *atpI*), 15 photosystem II subunits (*psbA*, *psbB*, *psbC*, *psbD*, *psbE*, *psbF*, *psbH*, *psbI*, *psbJ*, *psbK*, *psbL*, *psbM*, *psbN*, *psbT*, and *psbZ*), 12 NADH dehydrogenase subunits (*ndhA–ndhK*, with *ndhB* duplicated), six cytochrome b/f complex subunits (*petA*, *petB*, *petD*, *petG*, *petL*, and *petN*), five photosystem I subunits (*psaA*, *psaB*, *psaC*, *psaI*, and *psaJ*), and the Rubisco large subunit (*rbcL*). Self-replication genes included 11 large ribosomal subunit proteins (*rpl* genes), 13 small ribosomal subunit proteins (*rps* genes, with *rps7* duplicated), four DNA-dependent RNA polymerase subunits (*rpoA*, *rpoB*, *rpoC1*, and *rpoC2*), and four rRNA genes. Other genes included *accD* (acetyl-CoA carboxylase subunit), *ccsA* (cytochrome c synthesis), *cemA* (envelope membrane protein), *clpP* (protease), *infA* (translation initiation factor), and *matK* (maturase), as well as four conserved open reading frames (*ycf1*, *ycf2*, *ycf3*, and *ycf4*) and *ycf15* (present in two copies). A total of 22 genes contained introns, with 19 genes possessing a single intron and three genes (*clpP*, *ycf3*, and *rps12*) containing two introns (Table 5). Notably, *trnK-UUU* contained the largest intron, which included the *matK* gene, a typical feature of angiosperm chloroplast genomes.

**Table 5.** Lengths of exons and introns for the split genes in the *Hydrangea medogensis* chloroplast genome. Genes located in the inverted repeat (IR) regions are present in two copies (IRa and IRb) with opposite transcriptional orientations. Note: *rps12* is a trans-splicing gene with three exons dispersed at different locations.

**Gene**	**Strand**	**Start**	**End**	**Exon I**	**Intron I**	**Exon II**	**Intron II**	**Exon III**
*atpF*	−	12,544	13,816	152	685	436	−	−
*clpP*	−	72,296	74,354	71	772	288	726	202
*ndhA*	−	122,287	124,502	553	1,124	539	−	−
*ndhB*	−	96,999	99,210	775	679	758	−	−
*ndhB-copy2*	+	144,282	146,493	775	679	758	−	−
*petB*	+	77,263	78,674	6	758	648	−	−
*petD*	+	78,866	80,103	9	755	474	−	−
*rpl16*	−	83,550	84,967	9	1,010	399	−	−
*rpl2*	−	86,646	88,135	397	662	431	−	−
*rpl2-copy2*	+	155,357	156,846	397	662	431	−	−
*rpoC1*	−	21,808	24,656	451	767	1,631	−	−
*rps12*	−	72,044	100,837	232	536	26	27,886	114
*rps16*	−	5,143	6,271	39	865	225	−	−
*ycf3*	−	43,899	45,865	124	716	230	744	153

Additionally, several genes exhibited special characteristics: *psbL* possessed a non-standard start codon (ACG), *rps19* used GTG as the start codon, and *ndhD* used AAT as the start codon. Some multicopy genes showed length variation, such as marked length differences among *ycf1* copies (1,077–5,703 bp).

### IR/SC boundary analysis

**Figure 7.**
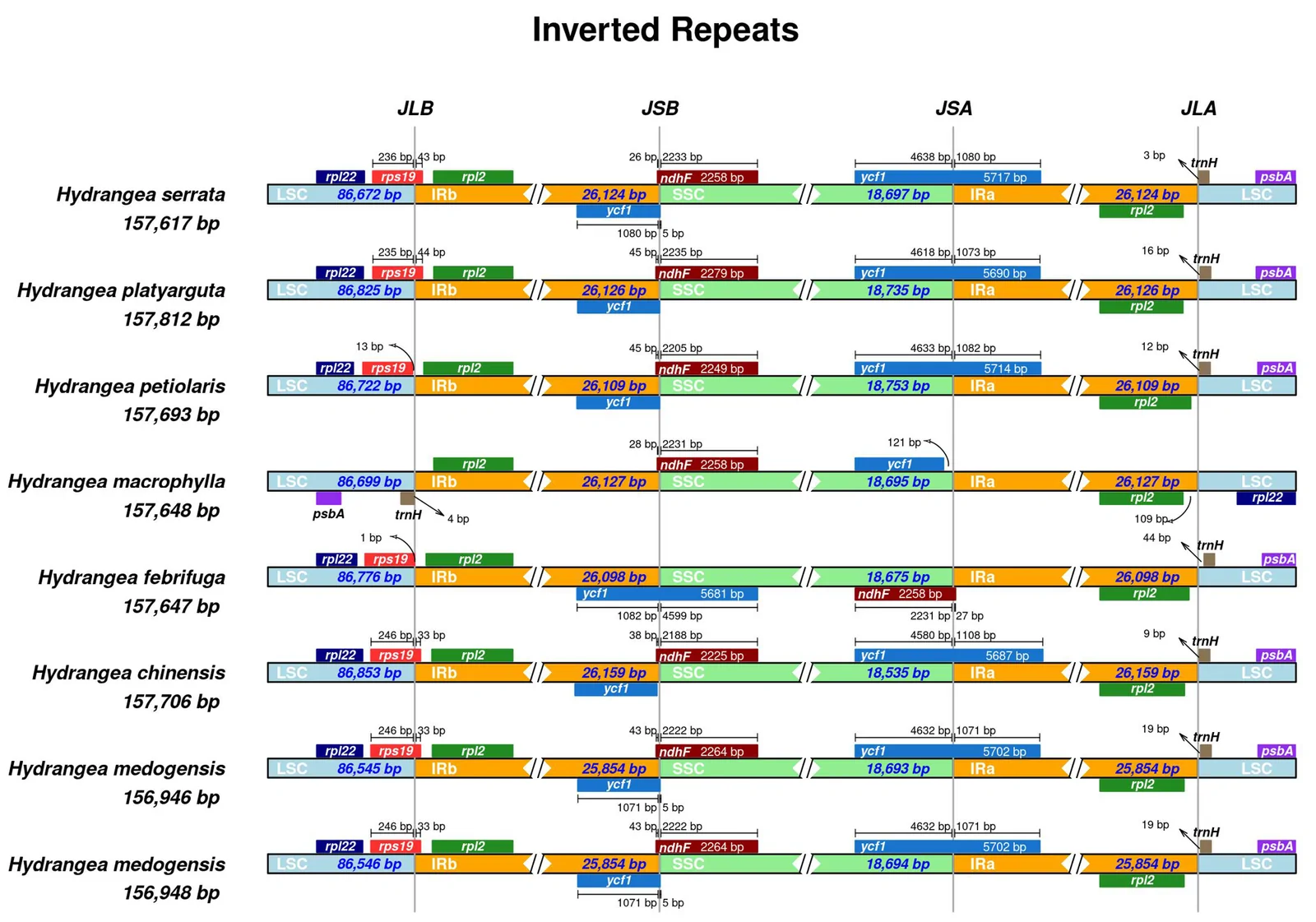
Comparison of the inverted repeat (IR)/single-copy (SC) boundary regions among *Hydrangea medogensis* and related *Hydrangea* species. The four junction sites are labeled as JLB (LSC–IRb), JSB (SSC–IRb), JSA (SSC–IRa), and JLA (LSC–IRa). Genes spanning the boundaries are shown with their respective lengths (bp) extending into adjacent regions. The diagram illustrates the expansion and contraction patterns of IR regions across the analyzed taxa.

The IR/SC boundary regions of *Hydrangea medogensis* were compared with those of six other *Hydrangea* species (*H. serrata*, *H. platyarguta*, *H. petiolaris*, *H. macrophylla*, *H. febrifuga*, and *H. chinensis*). The results showed that the boundary junction structures were generally conserved across all analyzed taxa but exhibited lineage-specific variations in gene cross-region lengths and precise junction positions (Fig. 7).

In *H. medogensis*, the LSC/IRb junction (JLB) was located within the *rps19* gene, with 246 bp of *rps19* extending into the LSC region and 33 bp extending into the IRb region. The SSC/IRb junction (JSB) was located within the *ndhF* gene, with 43 bp of *ndhF* extending into the SSC region and 2,222 bp extending into the IRb region. The SSC/IRa junction (JSA) was located within the *ycf1* gene, with 4,632 bp of *ycf1* extending into the SSC region and 1,071 bp extending into the IRa region. Due to the inverted repeat nature of the IRa and IRb regions, a portion of the *ycf1* fragment was duplicated in the IRb region and adjacent to *ndhF*. The LSC/IRa junction (JLA) was located between *trnH* and *rpl2*; the *rpl2* gene was positioned in the IRa region, and *trnH* was located in the LSC region.

The boundary positions of the two *H. medogensis* individuals were almost identical, with the LSC and SSC lengths differing by only 1–2 bp, confirming the high intraspecific stability of the IR/SC boundaries. Compared with other *Hydrangea* species, *H. medogensis* possessed the shortest total genome length (156,946–156,948 bp) and the smallest LSC region (86,545–86,546 bp), whereas the IR length (25,854 bp) was similar to that of the other *Hydrangea* species. These boundary positions were generally conserved in *Hydrangea*, with only minor differences in gene cross-region lengths.

## Discussion

Molecular phylogenetic results indicated that *Hydrangea medogensis* formed a distinct group within Clade 1, sister to the remainder of the clade. Although *H. medogensis* is phylogenetically distant from *H. stylosa*, the two species share certain morphological similarities, including shrub habit (ca. 1–1.5 m tall), grayish-white second-year branchlets, papery elliptic leaves with shortly acuminate apices and cuneate to subrounded bases, and corymbose cymes with truncate apices. These shared characters likely represent plesiomorphic traits retained in both lineages or convergent evolution in vegetative morphology adapted to similar understory habitats. However, *H. medogensis* can be readily distinguished from *H. stylosa* by several diagnostic characters: the adaxial leaf surface is covered with long and short hairs (vs. nearly glabrous or glabrous in *H. stylosa*), the ovary is more than 4/5 inferior (vs. ca. 2/3 inferior), the calyx tube is subglobose with a constricted opening (vs. campanulate with a tubular opening), the calyx teeth are triangular and 0.6–1 mm long (vs. ovate to suborbicular and 1–1.5 mm long), and the seeds are narrowly winged around the margin (vs. minutely winged at one or both ends).

The phylogenetic analysis (Fig. 5) revealed that *Hydrangea* could be divided into two major clades: Clade 1 and Clade 2 are reciprocal sister groups. Clade 1 mainly includes Sect. *Petalanthae*, whereas Clade 2 includes Sect. *Heteromallae*, Sect. *Calyptranthe*, Sect. *Cornidia*, and Sect. *Hydrangea*. *Hydrangea medogensis* was included in Clade 1 but was not assigned to Sect. *Petalanthae*, instead occupying a sister-group position to the rest of the sampled taxa of this clade. According to the grouping key of *Hydrangea* in Flora of China (Wei and Bartholomew 2001), the new species possesses general characteristics of *Hydrangea* s.str., but its ovary position (more than 4/5 inferior) falls in a transitional state between the two primary branches of the key and thus cannot be accurately assigned to either the “completely inferior” or “1/3–2/3 superior” category. If temporarily placed under the branch of 1/3–2/3 superior ovary, the truncate base of petals, reticulate seed venation with a very narrow wing, and other features of the new species do not match the diagnostic characteristics of Sect. *Petalanthae* or Sect. *Heteromallae*: Sect. *Petalanthae* has free petals usually clawed at the base and seeds usually wingless or with very short wings; Sect. *Heteromallae* has seeds with long wings at both ends and unequal stamens, with the longer ones inflexed in bud. Conversely, if placed under the branch of completely inferior ovary, the new species is a shrub with free petals that do not fall off during anthesis, and its seeds with long reticulate venation and a very narrow wing around the margin, as well as the absence of bracts, are also inconsistent with the diagnostic characteristics of Sect. *Calyptranthe* and Sect. *Hydrangea*: Sect. *Calyptranthe* comprises climbing shrubs with petals connate into a caducous calyptrate corolla; Sect. *Hydrangea* has seeds with longitudinal venation and wings at both ends and possesses bracts that are usually lanceolate.

In conclusion, *H. medogensis* occupies a transitional morphological position within the existing sectional classification system of *Hydrangea* s.str. Its unique ovary position and floral and seed characteristics preclude its assignment to any known section. Therefore, the new species is provisionally assigned to *Hydrangea* without a sectional affiliation, and its exact taxonomic position awaits further determination based on more comprehensive sampling and genomic data.

In addition, it should be noted that only ITS sequences were used in the phylogenetic analysis of the present study. Although plastid markers are valuable for resolving deeper nodes and a combination of different markers is generally preferable, the inclusion of plastid sequences was constrained by the extremely limited molecular data available for closely related species, particularly *H. stylosa*, which is morphologically most similar to the new species. A survey of public databases revealed that *H. stylosa* is currently represented by only a few short and fragmentary plastid sequences, with no complete chloroplast genome or low-copy nuclear gene sequences available. Furthermore, many of these plastid fragments from *H. stylosa* lack sufficient overlap with homologous regions in other *Hydrangea* species, precluding the construction of a reliable concatenated matrix. Therefore, the phylogenetic tree was tentatively constructed based on ITS sequences to preliminarily assess the phylogenetic position of the new species. However, the relatively short length of the ITS region provides limited phylogenetic information, which may account for the relatively low support values observed for some branches. Future studies employing whole chloroplast genomes and additional nuclear markers are necessary to more precisely resolve the phylogenetic relationships of *H. medogensis* within *Hydrangea*.

## Supplementary Material

XML Treatment for Hydrangea medogensis

## Additional information

### Artificial Intelligence (AI) use

The authors accept full responsibility for the content of the manuscript, including the disclosure of any use of AI.

The following AI tools were used in the preparation of this manuscript:

**AI tools**: Kimi.

**Used for**: Language, style and writing.

**Description**: The authors used AI tools for language editing and translation assistance during the preparation of this manuscript. All authors reviewed and approved the final content.

### Author ORCIDs

Wen-Qi He https://orcid.org/0009-0008-3041-5422

Fang-Yu Zhao https://orcid.org/0000-0001-6352-2955

Kai-Xin Yin https://orcid.org/0009-0001-5939-6797

Lian-Qiang Li https://orcid.org/0009-0009-2149-6897

Kai-Hui Zhao https://orcid.org/0000-0002-6665-2648

Fang-yuan Zhang https://orcid.org/0000-0003-3840-9947

Zhi-hua Liao https://orcid.org/0000-0002-1283-4649

Xiao-Zhong Lan https://orcid.org/0000-0002-8657-5790

### Data availability

All of the data that support the findings of this study are available in the main text.
